# Hyperspectral Imaging for Objective Assessment of Skin Prick Test Reactions: A Single-Subject Proof-of-Concept Study of Interobserver Reliability and Spectral Index Separation

**DOI:** 10.3390/diagnostics16193085

**Published:** 2026-09-23

**Authors:** Adrian Luehring, Maximilian Lammer, Cornelia Gattringer, Paul Bellmann, Rohit Arora, Miranda Klosterhuber, Lukas Kampik, Christian Wolfgang Huck, Johannes Dominikus Pallua

**Affiliations:** 1 Department of Orthopaedics and Traumatology, Medical University of Innsbruck, Anichstraße 35, 6020 Innsbruck, Austria; adrian.luehring@i-med.ac.at (A.L.); rohit.arora@i-med.ac.at (R.A.); miranda.klosterhuber@i-med.ac.at (M.K.); lukas.kampik@i-med.ac.at (L.K.); 2 Department of Dermatology, Venereology and Allergy, Medical University of Innsbruck, 6020 Innsbruck, Austria; maximilian.lammer@i-med.ac.at (M.L.); cornelia.gattringer@tirol-kliniken.at (C.G.); paul.bellmann@i-med.ac.at (P.B.); 3 Institute of Analytical Chemistry and Radiochemistry, University of Innsbruck, Innrain 80–82, 6020 Innsbruck, Austria; christian.w.huck@uibk.ac.at

**Keywords:** hyperspectral imaging, skin prick test, allergy, interobserver reliability, intraclass correlation coefficient, tissue oxygenation, proof of concept, baseline-corrected area under the curve

## Abstract

**Background/Objectives:** Hyperspectral imaging (HSI) enables non-contact assessment of tissue oxygenation and perfusion-related properties and may provide objective information beyond conventional visual assessment of skin prick test (SPT) reactions. This proof-of-concept study evaluated the interobserver reliability and within-subject spectral separation of HSI-derived indices during SPT. **Methods:** One healthy adult volunteer underwent standardised SPT on two separate days with histamine as the positive control, NaCl as the negative control, normal skin, and a non-reactive allergen site. HSI was acquired at 0, 5, 10, 15, and 45 min using a Tivita^®^ Tissue camera. Three raters independently assessed four predefined regions of interest. Five indices were analysed: near-infrared reflectance (NIR), tissue oxygen saturation (StO_2_), tissue haemoglobin index (THI), tissue water index (TWI), and tissue lipid index (TLI). Interobserver reliability was assessed using ICC(3,1), and within-subject separation was described using baseline-corrected trapezoidal area under the curve (AUCbc) and delta-AUCbc values. **Results:** StO_2_ showed the most consistent interobserver reliability, with good-to-excellent ICCs across all four ROIs at both visits. THI showed excellent reliability at the histamine and NaCl sites, whereas NIR showed excellent reliability at the histamine site and good reliability at the NaCl site. Reliability at the less clearly defined control sites was more variable across indices and visits. StO_2_ provided the largest cumulative separation of the histamine response from all controls (mean delta-AUCbc: +1204 vs. NaCl, +1300 vs. normal skin, and +1238 vs. non-reactive allergen; index-units × min). THI showed secondary separation, NIR moderate separation, TWI marginal separation, and TLI an inverse response. **Conclusions:** HSI, particularly StO_2_, with complementary information from THI and NIR, showed reproducible within-subject spectral changes during SPT. These exploratory findings support prospective validation in a larger multi-subject, multi-allergen cohort.

## 1. Introduction

The skin prick test (SPT) remains the cornerstone of type I hypersensitivity diagnosis, offering simplicity, rapid results, and broad allergen coverage [1]. However, conventional assessment relies on subjective measurement of wheal diameter and erythema area, which is influenced by inter-rater variability, operator experience, and the inherent difficulty of quantifying irregular wheals [2,3]. Published inter-operator Coefficients of Variation for SPT wheal measurement reach 20% or higher, undermining the test’s utility for longitudinal monitoring and clinical research [4].

Previous optical approaches have already demonstrated that infrared and hyperspectral imaging can provide objective information on skin-test reactions. Tversky and MacGlashan used short-wave infrared (SWIR) imaging centred at 1400 ± 100 nm, exploiting the strong water sensitivity of this spectral region to delineate oedematous wheals; in 24 individuals, SWIR detected wheals with 100% sensitivity and showed close agreement with standard manual measurements (Spearman R_s_ = 0.960) [5]. Nishino et al. subsequently demonstrated that long-wavelength near-infrared HSI (>1000 nm) could distinguish histamine-induced type I reactions from normal skin and type IV contact hypersensitivity using canonical discriminant analysis, with classification accuracies of 87.0% for type I, 87.67% for normal skin, and 98.5% for type IV reactions [6]. These spectral ranges probe partly different physiological mechanisms: SWIR wavelengths around 1400 nm are predominantly sensitive to tissue water and oedema, whereas the visible-to-near-infrared range used in the present study is more strongly influenced by haemoglobin absorption, oxygenation, and related microcirculatory changes.

Hyperspectral imaging (HSI) is a non-contact optical imaging technique that captures spatially resolved reflectance spectra in the visible to near-infrared range and enables the assessment of microcirculatory parameters, including superficial tissue oxygenation, near-infrared perfusion, haemoglobin distribution, and tissue water content [7].

The technology has been evaluated across a growing range of clinical applications, including wound and burn assessment, peripheral and perioperative microcirculatory monitoring, bowel perfusion, and intraoperative tissue characterization [8,9,10,11,12,13,14].

In the context of cutaneous inflammation, HSI captures physiological correlates of wheal-flare reactions—vascular dilatation, plasma extravasation, and reactive hyperaemia—that may be objectively quantified before macroscopic changes become visible [15].

These prior studies establish the feasibility of optical and hyperspectral assessment of allergic skin reactions, but they focused primarily on wheal delineation in the SWIR range or spectral classification of allergic reaction types. To our knowledge, no previous study has combined a clinically available visible-to-near-infrared HSI system with serial multi-index quantification, multiple control conditions, and formal interobserver reliability assessment during SPT. Key open questions include (1) which spectral indices reliably differentiate histamine-positive from control skin conditions; (2) what level of interobserver reliability can be achieved for HSI index measurements derived from independently placed regions of interest (ROIs); and (3) whether spectral responses are temporally consistent across observation windows.

We present a proof-of-concept single-subject study using the Tivita^®^ Tissue camera system, evaluating five HSI indices (NIR, StO_2_, THI, TWI, and TLI) at five time points, across four ROIs, and with three raters during two separate visits. The primary objectives were to generate preliminary estimates of interobserver reliability for ROI-derived HSI index measurements and to characterise within-subject spectral separation patterns using descriptive baseline-corrected AUC (AUCbc) analysis, thereby providing planning data for a subsequent adequately powered clinical validation study.

## 2. Materials and Methods

### 2.1. Study Design and Participants

This retrospective single-subject proof-of-concept study analysed repeated hyperspectral measurements obtained during two clinically indicated allergological examinations (T1 and T2). The analysis was designed to assess technical feasibility, interobserver reliability of ROI-derived HSI index measurements, and within-subject spectral response patterns rather than to provide population-level diagnostic estimates. The participant was a healthy adult volunteer with no history of atopic disease, allergic rhinitis, or current antihistamine use. Written informed consent for the retrospective scientific analysis and anonymised publication was obtained. No additional study-specific procedures were performed. The underlying allergological examinations were clinically indicated, and hyperspectral imaging was performed as complementary documentation using a CE-marked, non-invasive, contact-free imaging system. According to the institutional regulations of the Medical University of Innsbruck for retrospective single-case analyses and case series of up to five cases, formal submission to the Ethics Committee was not required. The Ethics Committee of the Medical University of Innsbruck confirmed that formal ethical review was not required for the present analysis.

### 2.2. Skin Prick Test Protocol

Standard SPT was performed on the volar forearm using a lancet device as part of the routine clinical allergological examination. Test substances included histamine dihydrochloride (10 mg/mL) as a positive control, 0.9% NaCl as a negative control, and clinically indicated allergen extracts. Because the present study was a retrospective analysis of routinely performed clinical examinations, product-specific manufacturer information for the lancet device, histamine solution, saline solution, and allergen extracts was not available from the retrospective documentation. For the present analysis, one allergen application site without visible clinical reaction was selected as an additional non-reactive control site. Because the study was designed as a single-subject methodological proof of concept rather than an allergen-specific comparison, a single representative non-reactive allergen site was included to provide an additional control condition without introducing multiple correlated allergen-control sites from the same examination. Application sites were marked with permanent ink and maintained at a standardised inter-site distance (≥3 cm). No antihistamines were administered within 5 days before testing.

### 2.3. Hyperspectral Imaging Acquisition

HSI was performed using the Tivita^®^ Mobile hyperspectral imaging system (Diaspective Vision GmbH, Pepelow, Germany), acquiring 100 spectral bands across the 500–1000 nm range at a spatial resolution of 640 × 480 pixels. Hyperspectral images were acquired at five predefined time points: immediately before application of histamine, allergen, or control solution (baseline, t = 0), and at 5, 10, 15, and 45 min after prick application. As the Tivita^®^ system natively outputs only integer index values, the t = 10 min measurement was computed as the arithmetic mean of acquisitions at t = 7 and t = 13 min, rounded to the nearest integer. Acquisition was performed in a standardised setting (constant ambient light, patient immobilisation, fixed camera-to-skin distance of approx. 50 cm). Five proprietary spectral indices were derived: near-infrared reflectance (NIR), tissue oxygen saturation (StO_2_), tissue haemoglobin index (THI), tissue water index (TWI), and tissue lipid index (TLI). Although OHI was additionally available as a native TIVITA^®^ output, it was not included in the quantitative analyses.

### 2.4. Region of Interest Placement and Raters

Three trained raters, blinded to each other’s annotations, independently placed circular ROIs on all four skin conditions at each time point and visit. ROI size was not fixed but was individually adapted by each rater to the visible extent of the respective local skin reaction using the marker tool implemented in the TIVITA^®^ 2.0 Suite (version 2.0; Diaspective Vision GmbH, Pepelow, Germany). Marker size was adjusted via the software-defined cursor-size setting, with settings ranging from 5 to 20 in the present analysis. Thus, both ROI position and ROI size represented observer-dependent components of the measurement procedure. ROI-level index values were extracted separately for each rater, time point, skin condition, and visit. ROI placement was standardised using anatomical landmarks and ink markings but was performed without reference to the other raters’ placements. Interobserver agreement was subsequently assessed on the quantitative HSI index values derived from these independently placed ROIs rather than on the geometric overlap of the ROI annotations themselves.

### 2.5. Statistical Analysis

All statistical analyses were considered exploratory and descriptive due to the single-subject proof-of-concept design. Given *n* = 1 biological subject, no inferential statistics were applied to within-subject spectral separation; all comparisons are purely descriptive.

Interobserver reliability of ROI-derived HSI index measurements was quantified using a two-way mixed-effects intraclass correlation coefficient for single measurements with absolute agreement [ICC(3,1)], following Koo and Li (2016) [16].

For each HSI index, ROI, and visit, ICC(3,1) was calculated across the repeated measurement time points, with the measurement time points treated as targets and the three observers treated as raters. The ICC therefore quantifies agreement in ROI-derived HSI index values between observers rather than spatial agreement of ROI placement. ICC values were interpreted as <0.50, poor; 0.50–0.74, moderate; 0.75–0.89, good; and ≥0.90, excellent. Negative ICC estimates were retained as calculated and interpreted as indicating no reliable interobserver agreement.

Descriptive spectral separation across conditions was quantified using the baseline-corrected area under the curve (AUCbc) over the post-baseline observation period from 5 to 45 min. AUCbc was calculated for each hyperspectral index, skin condition, and study session using the arithmetic mean across all three raters. For baseline correction, the value at t = 0 was subtracted from each subsequent time point before integration, thereby quantifying the net temporal change from baseline rather than the absolute index level. The integrated response was estimated using the linear trapezoidal method, which approximates the cumulative signal change by summing the areas between consecutive time points. To assess the cumulative magnitude of histamine-induced spectral separation, delta-AUCbc values were calculated as the difference between the positive histamine control and each respective control condition. All AUCbc and delta-AUCbc values are reported in index-units × min and were interpreted descriptively, without inferential statistical testing, owing to the single-subject proof-of-concept design. Analyses were performed in R version 4.5.1 (R Foundation for Statistical Computing, Vienna, Austria; https://www.r-project.org/) using the packages pracma 2.4.6 (https://CRAN.R-project.org/package=pracma, accessed on 11 August 2026) and irr 0.85 (https://CRAN.R-project.org/package=irr, accessed on 11 August 2026).

## 3. Results

### 3.1. Interobserver Reliability of ROI-Derived HSI Index Measurements

ICC(3,1) estimates of interobserver reliability for ROI-derived HSI index measurements across all five indices, four ROIs, and two visits are summarised in Table 1 and visualised in Figure 1. Reliability varied by both spectral index and ROI location. StO_2_ demonstrated the most consistent reliability profile, achieving good-to-excellent reliability across all ROIs at both visits (ICC 0.758–0.997). THI showed excellent reliability at ROIs 1 (positive histamine) and 2 (negative NaCl) at both visits, whereas NIR showed excellent reliability at ROI 1 and good reliability at ROI 2. Reliability at the less clearly defined control ROIs was more variable. For NIR, ROI 3 showed poor reliability at both visits, whereas ROI 4 improved from poor reliability at T1 (ICC = −0.156) to excellent reliability at T2 (ICC = 0.928). THI showed poor reliability at ROI 3 at both visits, while ROI 4 improved from poor reliability at T1 (ICC = 0.098) to good reliability at T2 (ICC = 0.868). TLI showed excellent reliability at ROIs 1 and 2 at both visits and poor reliability at ROI 3, whereas ROI 4 showed good reliability at T1 and excellent reliability at T2. TWI showed good-to-excellent reliability at ROIs 1, 2, and 4, while ROI 3 increased from poor reliability at T1 (ICC = 0.308) to excellent reliability at T2 (ICC = 0.916). Overall, ROIs 1 and 2 showed the most consistently high interobserver reliability across indices and visits, whereas reliability at ROIs 3 and 4 was more index- and visit-dependent. Negative ICC estimates (e.g., NIR at ROI 3 in T2, ICC = −0.318) were interpreted as indicating an absence of reliable interobserver agreement rather than meaningful negative reliability; such estimates can occur when within-target disagreement or measurement error exceeds the variability between measurement targets.

### 3.2. Descriptive AUCbc Analysis of Within-Subject Spectral Separation

To characterise the cumulative temporal magnitude of spectral separation between conditions, baseline-corrected trapezoidal AUC (AUCbc) values were computed over t = 5–45 min for each HSI index, condition, and session (mean across all three raters). Delta-AUCbc values (positive histamine minus each control) provide a descriptive summary of the integrated net spectral response above baseline.

StO_2_ demonstrated the largest and most consistent cumulative net response at the histamine site. In T1, the positive histamine site yielded AUCbc = +1039.2 StO_2_-% × min, compared with −485.0 (neg_NaCl), −164.2 (normal_skin), and −383.3 (neg_allergen)—delta-AUCbc values of +1524.2, +1203.3, and +1422.5, respectively. In T2, the histamine AUCbc was +1125.0 vs. controls of +241.7, −270.8, and +70.8—delta-AUCbc of +883.3, +1395.8, and +1054.2. Across both sessions, mean delta-AUCbc for StO_2_ was +1203.8 vs. neg_NaCl, +1299.6 vs. normal_skin, and +1238.3 vs. neg_allergen (all index-units × min).

THI provided secondary cumulative net separation, particularly against NaCl and normal skin controls. In T1, delta-AUCbc was +960.0 vs. neg_NaCl, +853.3 vs. normal_skin, and +861.7 vs. neg_allergen. In T2, the delta-AUCbc against neg_allergen contracted to +549.2, with +504.2 vs. neg_NaCl and +721.7 vs. normal_skin, suggesting broadly maintained but session-variable separation that warrants attention in future multi-subject designs.

NIR showed moderate and broadly consistent cumulative net separation. T1 delta-AUCbc values were +278.3 vs. neg_NaCl, +384.2 vs. normal_skin, and +320.0 vs. neg_allergen; T2 values were +243.3, +312.5, and +230.0, respectively. The smaller absolute delta-AUCbc values reflect index-specific scaling rather than weaker discrimination. NIR reflectance in the 700–900 nm range is sensitive to multiple tissue optical properties, including scattering and water-related effects. The observed NIR response may therefore reflect tissue changes associated with histamine-induced wheal formation, including oedema, although the relative contribution of individual optical mechanisms cannot be determined from the present data.

TWI showed only marginal cumulative net separation in T1 (delta-AUCbc: +144.2 vs. neg_NaCl; +89.2 vs. normal_skin; +114.2 vs. neg_allergen) and near-null or negative separation in T2 (+14.2, +18.3, −1.7 respectively), indicating limited discriminative capacity for this index. TLI demonstrated consistent inverse directionality, with lower cumulative baseline-corrected values at the positive histamine site than at most control conditions. This pattern may be compatible with oedema-related changes in the relative contribution of water- and lipid-sensitive spectral features; however, the underlying mechanism cannot be established from the present single-subject data.

### 3.3. Temporal Dynamics and Index Trajectories

The distribution of HSI index values across skin conditions is illustrated as baseline-corrected trajectory plots in Figure 2 (baseline-corrected Δ-values per timepoint, T1 vs. T2) and as AUCbc bar plots in Figure 3 (cumulative baseline-corrected AUC per index, session, and condition). StO_2_, NIR, and THI exhibited the clearest net separation of positive histamine from controls across the observation window (t = 0–45 min), consistent with persistent vasodilation and oedema at the histamine wheal.

The StO_2_ trajectory shows a rapid rise from baseline to t = 5 min at the histamine site, followed by sustained elevation through t = 45 min, while all control ROIs remained comparatively stable. This pattern is visible in both T1 and T2, though the absolute level of the histamine response was higher in T2. THI and NIR exhibited similarly pronounced responses, with temporal trajectories broadly paralleling the StO_2_ response and showing clear separation of the histamine-positive site from the control ROIs. TWI showed limited and inconsistent divergence between conditions. TLI showed the expected inverse pattern—a relative decrease at the histamine site.

The spectral characteristics underlying the StO_2_ and NIR findings are further illustrated in Figure 4 and Figure 5. At t = 5 min, the positive histamine ROI (Marker 1) showed higher relative absorption in the 500–600 nm region than the three control ROIs. This wavelength range contains prominent haemoglobin-sensitive spectral features and is therefore compatible with altered local haemoglobin-related optical properties during the histamine response. Differences in the NIR range may additionally reflect changes in tissue optical properties associated with the developing wheal. Higher NIR reflectance (700–1000 nm) is consistent with increased tissue scattering from subepidermal oedema fluid.

Figure 6 provides a spatially resolved, qualitative illustration of the StO_2_ dynamics underlying the quantitative AUCbc separation reported above. At baseline (t = 0), the false-colour StO_2_ distribution across the volar forearm was largely homogeneous, with all four ROIs (Markers 1–4) displaying comparable low-to-intermediate oxygenation and no focal signal at the prospective histamine site. From t = 5 min onward, a well-demarcated high-StO_2_ focus (red) emerged at the histamine wheal (Marker 1), spatially concordant with the ink-marked prick location and consistent with H_1_-receptor-mediated vasodilation and reactive hyperaemia. This focal oxygenation signal intensified and expanded through t = 10–15 min, reaching its greatest spatial extent and colour intensity at the mid-observation window, and remained visibly elevated at t = 45 min, indicating a sustained rather than transient response. The three control ROIs—negative NaCl (Marker 2), normal skin (Marker 3), and the non-reactive allergen site (Marker 4)—showed no comparable focal elevation and stayed within the baseline colour range across all time points, with only minor diffuse fluctuations attributable to physiological perfusion variability and slight inter-acquisition repositioning. The spatial coherence of the histamine hotspot with the anatomical prick site, together with the absence of an equivalent signal at control ROIs, supports the interpretation of the StO_2_ elevation as a localised inflammatory microcirculatory response rather than global signal drift or an acquisition artefact. Consistent with the single-subject proof-of-concept design, these observations are descriptive and illustrative: they visually corroborate the trajectory (Figure 2) and AUCbc (Figure 3, Table 2) findings but do not permit inferential generalisation.

## 4. Discussion

This proof-of-concept study extends earlier SWIR and long-wavelength NIR hyperspectral work on allergic skin reactions by evaluating a clinically available 500–1000 nm HSI platform with serial measurements, multiple spectral indices, and formal assessment of interobserver ROI reliability. Our findings suggest that HSI-derived spectral indices—particularly StO_2_, NIR, and THI—can capture reproducible within-subject spectral changes at histamine-challenged skin compared with multiple control conditions over a 45-min observation window.

Our results are complementary rather than directly equivalent to the earlier infrared studies. Tversky and MacGlashan demonstrated highly accurate wheal delineation around the water-sensitive 1400 nm region, whereas the Tivita^®^ system used here does not cover that wavelength range and instead derives physiological indices from 500–1000 nm data [5]. Nishino et al. showed that spectral information above 1000 nm can differentiate histamine-induced type I inflammation from type IV dermatitis and normal skin; however, their canonical discriminant classifier addressed reaction-type classification rather than longitudinal SPT quantification or ROI reliability [6]. The present data therefore suggest that haemoglobin- and oxygenation-sensitive information within the visible/NIR range may add a complementary physiological dimension to the predominantly water-sensitive SWIR assessment of wheal morphology.

### 4.1. Reliability of ROI-Derived HSI Index Measurements

The marked ROI dependence of interobserver agreement in ROI-derived HSI index values is one of the most clinically relevant findings of this study. ROIs 1 and 2, placed over the wheal centre and adjacent NaCl control, consistently achieved good-to-excellent reliability across indices and visits. By contrast, ROIs 3 and 4, located at neutral skin and allergen control sites with no hyperaemia, showed poor reliability across several indices. This likely reflects the absence of a visually or physiologically distinct target structure at control sites, which may increase variability in ROI placement and consequently in the derived HSI index values. Future prospective studies should therefore incorporate standardized ROI templates with predefined size and anatomically anchored placement criteria to minimize observer-dependent placement variability and improve reproducibility across measurements.

### 4.2. Spectral Index Performance

StO_2_ emerged as the primary discriminative index in this PoC study. The baseline-corrected AUCbc analysis confirmed substantially higher cumulative net oxygenation response at the histamine site compared with all three control conditions across both sessions. The temporal trajectory showed a rapid rise from baseline to t = 5 min, followed by sustained elevation through t = 45 min. This temporal pattern is compatible with the expected microcirculatory response to histamine, including vasodilation and reactive hyperaemia. Marker 1 showed higher relative absorption in the 500–600 nm haemoglobin-sensitive region than the control ROIs. This observation supports the presence of histamine-associated changes in local haemoglobin-related optical properties but does not allow direct quantification of oxyhaemoglobin concentration or identification of a single underlying physiological mechanism. This interpretation is consistent with the known local vascular effects of histamine, including H_1_-receptor-mediated vasodilation and increased cutaneous blood flow. In an allergen-induced type I hypersensitivity reaction, similar downstream vascular effects occur following mediator release from activated mast cells [9].

THI provided complementary discrimination, reflecting haemoglobin concentration changes consistent with oedematous wheal formation. The descriptive delta-AUCbc for THI was consistent against all three control conditions across sessions.

NIR demonstrated moderate and broadly consistent cumulative net separation from all three controls across both sessions. The smaller absolute delta-AUCbc values reflect index-specific scaling rather than weaker discrimination. NIR reflectance in the 700–900 nm range is sensitive to changes in tissue optical properties, including scattering and water-related effects. The observed NIR response may therefore reflect tissue changes associated with histamine-induced oedema and represents an optical signal complementary to the StO_2_ response. The moderate session-to-session variability may reflect partial oedema resolution or ROI-placement variability at the unmarked normal-skin site, which showed the lowest ICC reliability across all indices.

TWI showed only marginal cumulative net separation, with positive but small ΔAUCbc values in T1 and substantially attenuated or near-null separation in T2. Together with the observed session-dependent variability, these findings suggest that TWI has limited standalone utility for quantitative SPT assessment in the present proof-of-concept setting. Similar variability in the TIVITA-derived TWI has been reported during controlled vascular occlusion experiments, where the observed TWI response partly deviated from physiological expectations and the potential susceptibility of the underlying spectral bands to external influences was discussed [12].

TLI showed an inverse and comparatively variable response pattern. Although the reduction in TLI at the histamine site may be compatible with changes in the relative contribution of water- and lipid-sensitive spectral features during oedema formation, the present data do not permit a definitive physiological interpretation. Importantly, TLI did not provide a directionally consistent positive separation of the histamine reaction from the control conditions. TWI and particularly TLI should therefore currently be regarded as exploratory device-derived indices rather than robust standalone markers of SPT reactions. Their inclusion in the present analysis nevertheless provides a transparent comparison of the relative performance of the native TIVITA^®^ indices and may inform parameter selection in future prospective validation studies.

### 4.3. Limitations

This study is critically limited by its single-subject proof-of-concept design. With *n* = 1 biological subject, no inferential statistics can be generalised to a target population. All AUCbc and ΔAUCbc values are therefore purely descriptive and cannot establish population-level diagnostic performance, robust effect estimates, or inter-individual variability. Similarly, although repeated measurements across two visits and assessment by three independent raters provide information on within-subject measurement behaviour and interobserver agreement, they do not constitute independent biological replication. The observed temporal response patterns, spectral separation, and ICC estimates should therefore be considered participant-specific and should not be interpreted as population-level estimates of reproducibility. In addition, the wide ICC confidence intervals, inherent to the limited number of raters and measurement targets, preclude precise estimation of the corresponding population ICCs. External validity is consequently limited and requires confirmation in a prospectively powered multi-subject cohort.

The retrospective nature of the analysis represents an additional limitation. Although HSI acquisition was performed under a standardized clinical setup with constant ambient light, patient immobilisation, and an approximately fixed camera-to-skin distance, potentially relevant acquisition-related factors were not prospectively controlled or systematically documented with the rigor of a dedicated validation protocol. Subtle differences in ambient conditions, patient positioning, forearm orientation, skin preparation, ambient or skin temperature, and individual haemodynamic state between examinations may therefore have contributed to session-to-session variability.

Furthermore, the present single-subject design does not permit evaluation of physiological sources of inter-individual variability that may affect visible/NIR HSI-derived tissue parameters. In particular, skin pigmentation represents a potentially relevant optical confounder because melanin absorption overlaps with haemoglobin-sensitive spectral regions. Recent work using a 500–1000 nm TIVITA^®^ system demonstrated that skin pigmentation can influence HSI-derived tissue indices, emphasizing the need to account for skin tone in the interpretation and future validation of these parameters [17]. Future studies should therefore include participants with diverse skin pigmentation and prospectively evaluate relevant demographic and physiological covariates. Taken together, these limitations mean that the present findings should be interpreted exclusively as methodological proof-of-concept observations. Prospective multi-subject studies are required to determine inter-individual variability, establish population-level reliability and diagnostic performance, and assess the generalisability of the observed HSI response patterns.

### 4.4. Clinical Translation and Future Direction

The descriptive within-subject separations observed in this proof-of-concept study provide preliminary information for the design of a subsequent prospective validation study but do not permit a reliable sample-size calculation. The required sample size should therefore be determined prospectively using effect-size and variance estimates obtained from an independent multi-subject pilot cohort and based on a prespecified primary endpoint. A future validation study should employ a prospective cohort design with standardised imaging conditions, predefined ROI placement, blinded multi-rater assessment, and a standardised allergen panel to evaluate the diagnostic performance and reproducibility of HSI-based SPT quantification. Such multi-subject validation will be necessary to quantify inter-individual variability and determine whether the within-subject response patterns observed here are generalisable to the broader population.

## 5. Conclusions

This single-subject proof-of-concept study suggests that hyperspectral imaging can capture quantifiable spectral signatures of skin-prick test reactions from approximately 5 min post-application onward. Within the constraints of this single-subject design, StO_2_ emerged as the most informative and consistently reproducible index, with THI providing complementary haemoglobin-related information and NIR showing moderate and broadly consistent separation. By contrast, TWI and particularly TLI showed limited or variable discriminative behaviour and should currently be regarded as exploratory parameters for SPT assessment. These findings provide a methodological foundation and preliminary estimates of measurement behaviour that may inform the design and parameter selection of a prospectively powered multi-subject HSI-SPT validation study.

## Figures and Tables

**Figure 1:**
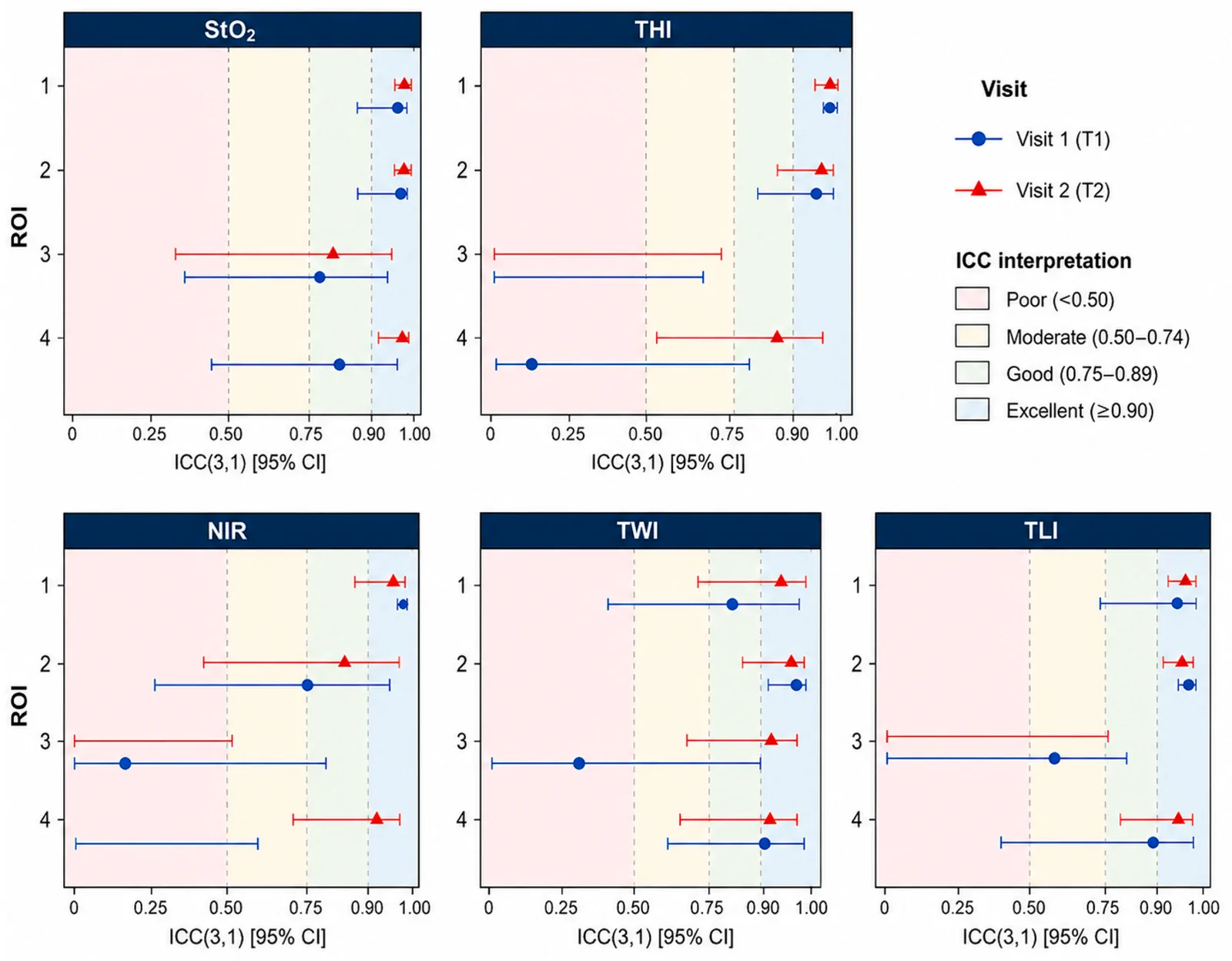
Interobserver reliability of ROI-derived HSI index measurements. Forest plots show ICC(3,1) estimates with 95% confidence intervals for five hyperspectral indices (StO_2_, THI, NIR, TWI, and TLI) across four predefined ROIs at Visit 1 (T1, blue circles) and Visit 2 (T2, red triangles). Background zones indicate reliability categories according to Koo and Li (2016) [16]: poor (<0.50), moderate (0.50–0.74), good (0.75–0.89), and excellent (≥0.90). ROI 1 = positive histamine; ROI 2 = negative NaCl; ROI 3 = normal skin; ROI 4 = non-reactive allergen site.

**Figure 2:**
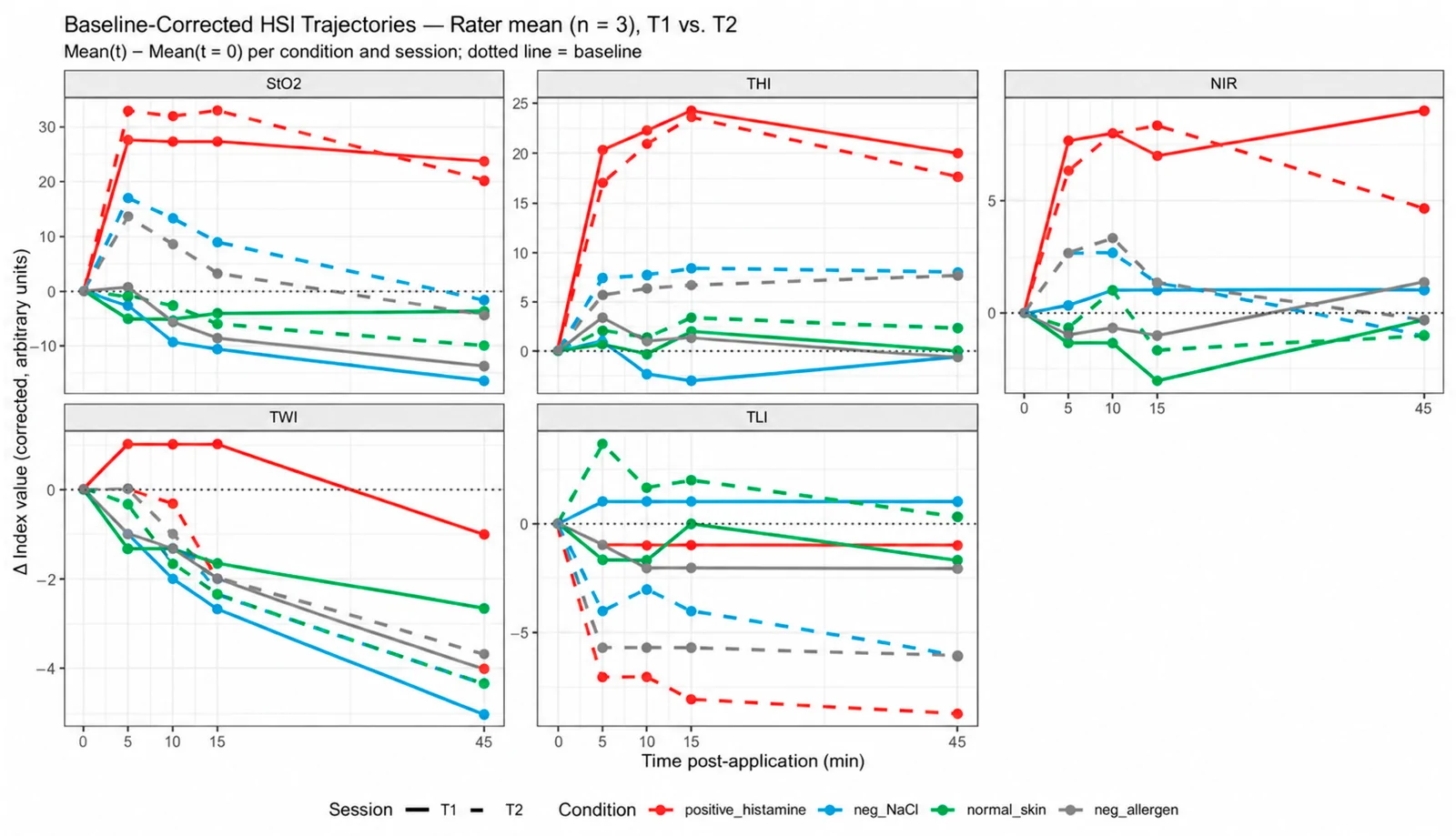
Baseline-corrected HSI index trajectories (T1 vs. T2). Δ-values (value at time point minus value at t = 0) over time (t = 0–45 min) per HSI index and skin condition. Solid lines = T1; dashed lines = T2.

**Figure 3:**
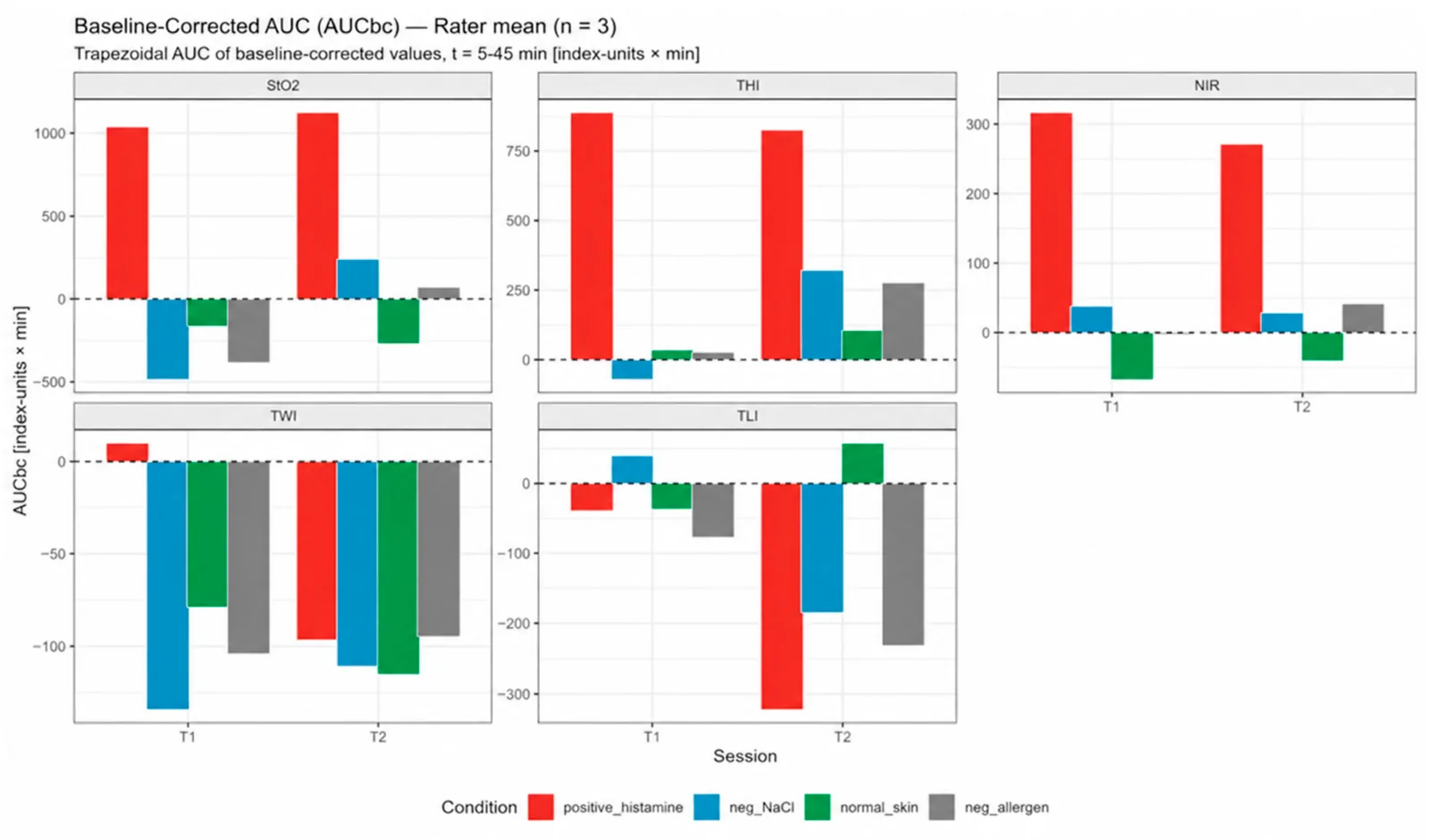
Baseline-corrected AUC (AUCbc) per HSI index, session, and condition. Trapezoidal AUCbc over t = 5–45 min (index-units × min), computed after subtracting the t = 0 baseline value. Bars represent cumulative net spectral response per condition across sessions T1 and T2.

**Figure 4:**
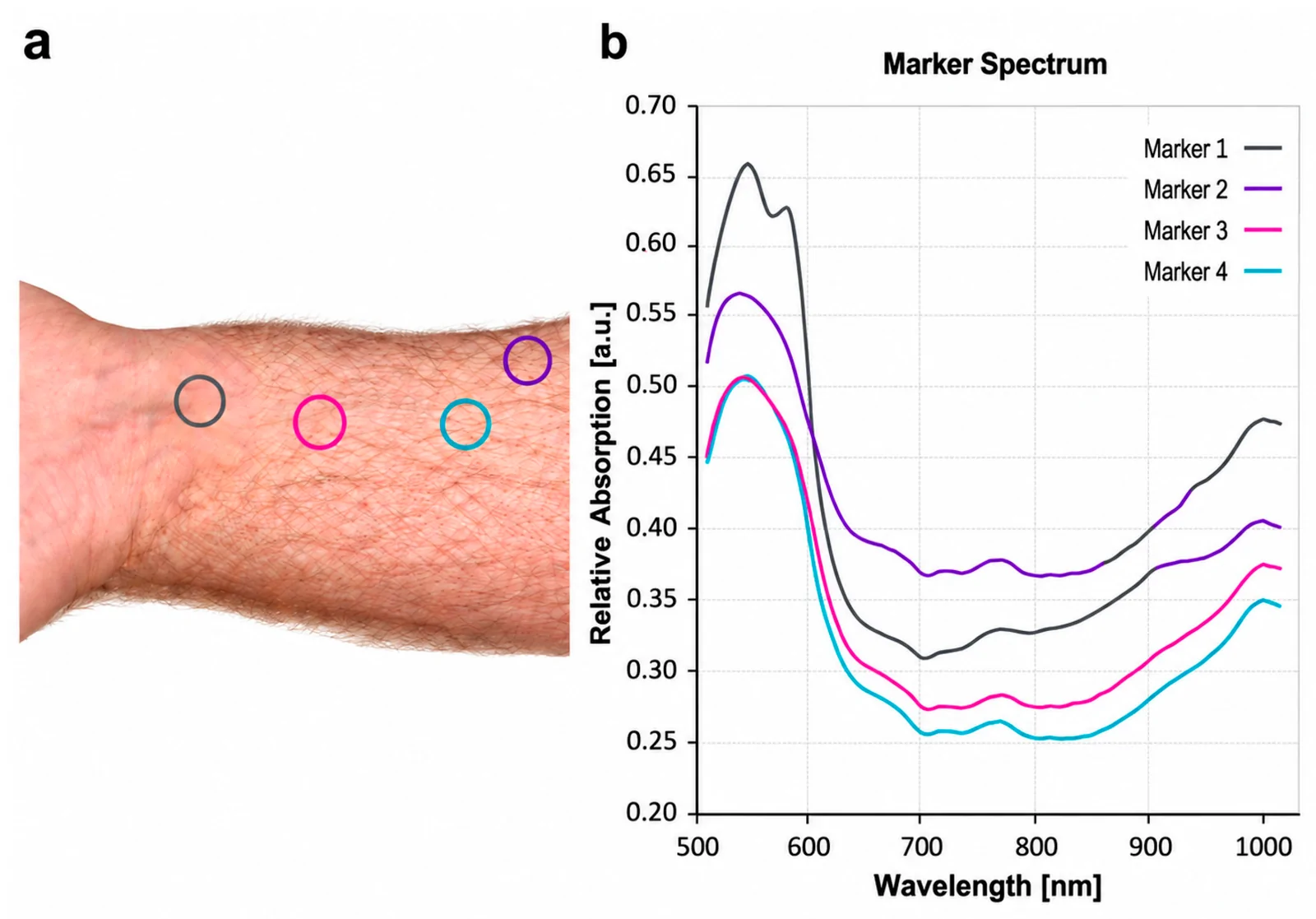
Representative hyperspectral imaging assessment of skin prick test sites at 5 min after application: (**a**) RGB image of the volar forearm showing four circular regions of interest (ROIs): Marker 1 (grey circle, positive histamine), Marker 2 (pink circle, negative NaCl), Marker 3 (purple circle, normal skin), and Marker 4 (cyan circle, non-reactive allergen site). (**b**) Corresponding mean relative absorption spectra from 500 to 1000 nm. The histamine-positive ROI shows higher relative absorption in the visible haemoglobin-sensitive region, particularly between approximately 500 and 600 nm, compared with the three control ROIs. Marker colours are consistent between the RGB image and the spectral profiles.

**Figure 5:**
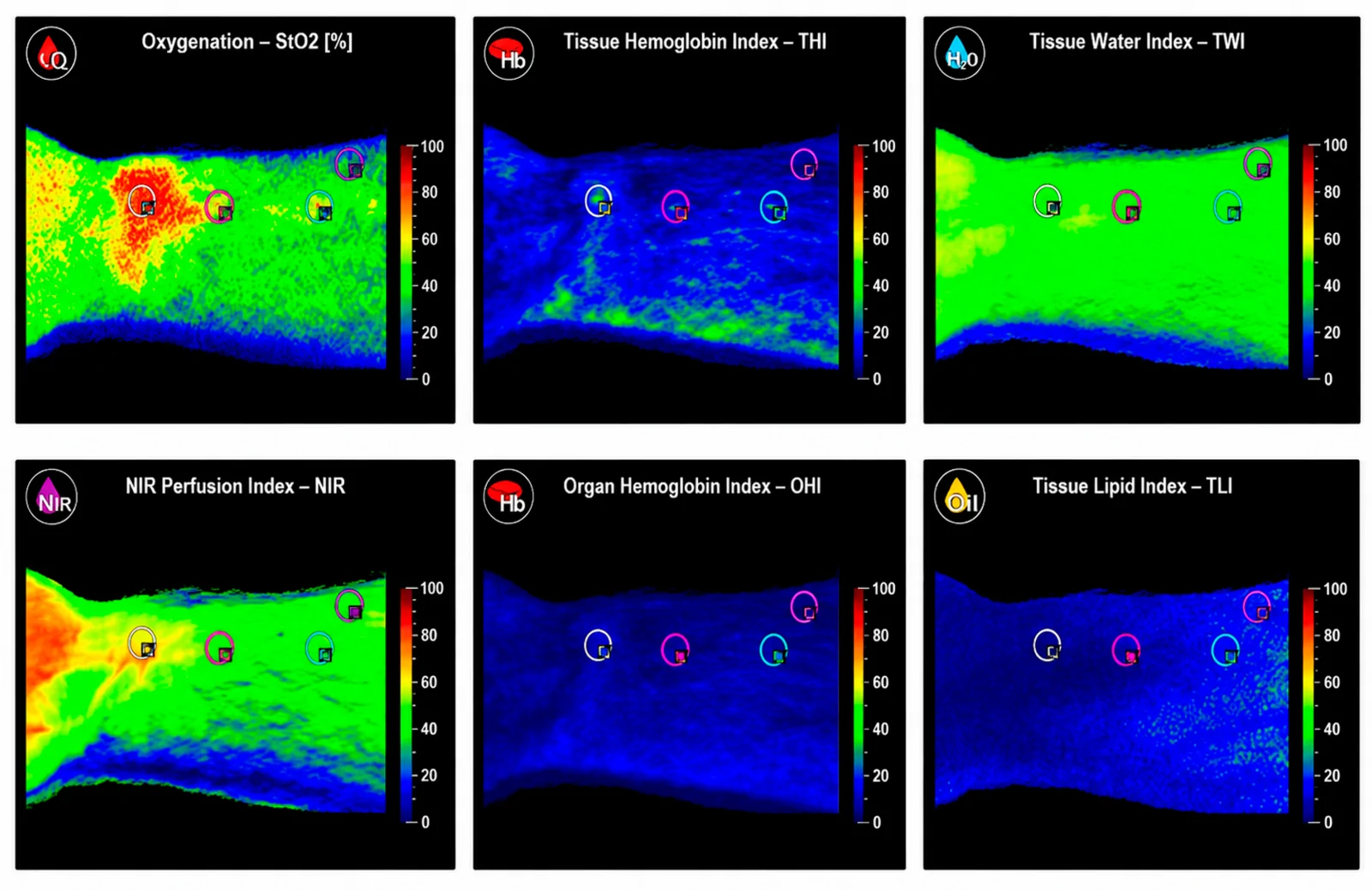
Simultaneous display of the six spectral index maps provided by the Tivita^®^ system: tissue oxygen saturation (StO_2_), tissue haemoglobin index (THI), and tissue water index (TWI) in the (**top row**), and near-infrared reflectance (NIR), organ haemoglobin index (OHI), and tissue lipid index (TLI) in the (**bottom row**). The colored circles indicate the four predefined measurement regions of interest (ROIs)/marker positions (Markers 1–4), which were kept identical across all spectral index maps. The maps are displayed using the native device-specific colour scales, with blue indicating lower and red indicating higher index values. OHI is shown for qualitative completeness as a native Tivita^®^ output but was not included in the quantitative statistical analyses.

**Figure 6:**
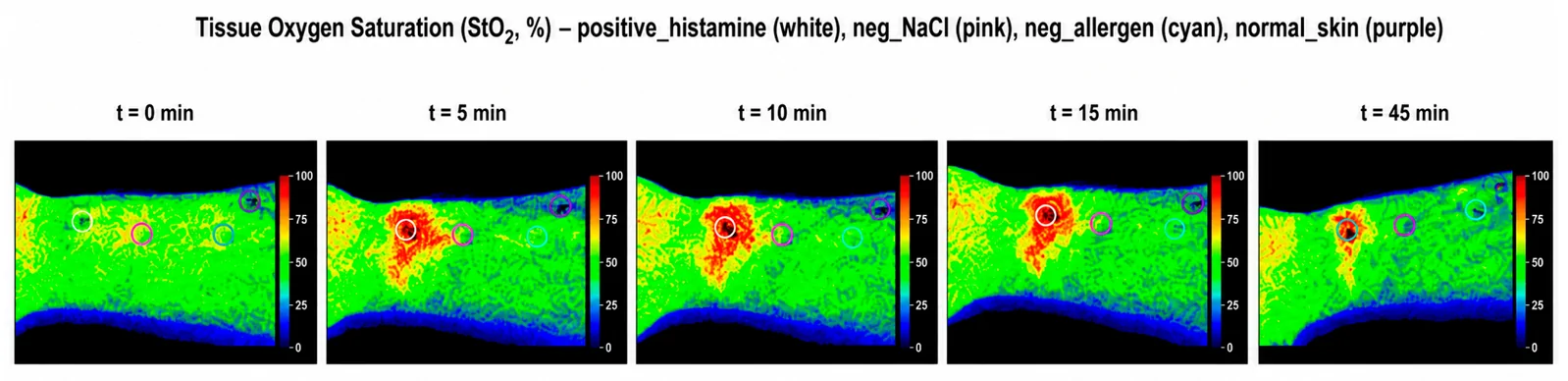
False-colour maps of tissue oxygen saturation (StO_2_, %) across all five measurement time points (t = 0, 5, 10, 15, and 45 min post-prick application). Marker 1 (grey circle = positive_histamine), Marker 2 (pink circle = negative NaCl), Marker 3 (purple circle = normal skin), Marker 4 (cyan circle = negative allergen). Colour scale: blue = low StO_2_, red = high StO_2_ (0–100%).

**Table 1:** Interobserver reliability of ROI-derived HSI index measurements assessed using ICC(3,1) with absolute agreement for five HSI indices across four ROIs (1: positive histamine, 2: negative NaCl, 3: normal skin, 4: non-reactive allergen site) and two visits. Reliability categories according to Koo and Li (2016) [16].

Index	ROI	T1 ICC	T1 95% CI	T1 Grade	T2 ICC	T2 95% CI	T2 Grade
NIR	1	0.995	0.978–0.999	Excellent	0.966	0.859–0.996	Excellent
	2	0.750	0.292–0.967	Good	0.843	0.435–0.981	Good
	3	0.174	−0.179–0.795	Poor	−0.318	−0.588–0.508	Poor
	4	−0.156	−0.429–0.596	Poor	0.928	0.704–0.992	Excellent
StO_2_	1	0.963	0.848–0.996	Excellent	0.991	0.960–0.999	Excellent
	2	0.995	0.978–0.999	Excellent	0.994	0.971–0.999	Excellent
	3	0.758	0.309–0.968	Good	0.789	0.283–0.973	Good
	4	0.812	0.409–0.976	Good	0.997	0.986–1.000	Excellent
THI	1	0.995	0.976–0.999	Excellent	0.992	0.962–0.999	Excellent
	2	0.953	0.806–0.995	Excellent	0.968	0.868–0.996	Excellent
	3	−0.101	−0.421–0.658	Poor	−0.042	−0.455–0.727	Poor
	4	0.098	−0.272–0.770	Poor	0.868	0.535–0.984	Good
TLI	1	1.000	1.000–1.000	Excellent	0.993	0.970–0.999	Excellent
	2	1.000	1.000–1.000	Excellent	0.997	0.985–1.000	Excellent
	3	0.491	−0.050–0.918	Poor	0.021	−0.473–0.774	Poor
	4	0.782	0.351–0.972	Good	0.978	0.906–0.997	Excellent
TWI	1	0.786	0.359–0.972	Good	0.920	0.689–0.991	Excellent
	2	0.982	0.921–0.998	Excellent	0.962	0.827–0.996	Excellent
	3	0.308	−0.138–0.860	Poor	0.916	0.663–0.990	Excellent
	4	0.887	0.601–0.986	Good	0.897	0.618–0.988	Good

**Table 2:** Descriptive baseline-corrected trapezoidal AUCbc (index-units × min) over t = 5–45 min per HSI index, session, and skin condition, with delta-AUCbc (Δ = positive_histamine − control). Green shading = positive delta (histamine > control); red shading = negative delta (histamine < control). Mean row = arithmetic mean of T1 and T2.

Index	Session	Pos. Histamine	Neg. NaCl	Normal Skin	Neg. Allergen	Δ vs. NaCl	Δ vs. Normal	Δ vs. Allergen
StO_2_	T1	+1039.2	−485.0	−164.2	−383.3	+1524.2	+1203.3	+1422.5
	T2	+1125.0	+241.7	−270.8	+70.8	+883.3	+1395.8	+1054.2
	Mean	+1082.1	−121.7	−217.5	−156.2	+1203.8	+1299.6	+1238.3
THI	T1	+888.3	−71.7	+35.0	+26.7	+960.0	+853.3	+861.7
	T2	+826.7	+322.5	+105.0	+277.5	+504.2	+721.7	+549.2
	Mean	+857.5	+125.4	+70.0	+152.1	+732.1	+787.5	+705.4
NIR	T1	+316.7	+38.3	−67.5	−3.3	+278.3	+384.2	+320.0
	T2	+271.7	+28.3	−40.8	+41.7	+243.3	+312.5	+230.0
	Mean	+294.2	+33.3	−54.2	+19.2	+260.8	+348.3	+275.0
TWI	T1	+10.0	−134.2	−79.2	−104.2	+144.2	+89.2	+114.2
	T2	−96.7	−110.8	−115.0	−95.0	+14.2	+18.3	−1.7
	Mean	−43.3	−122.5	−97.1	−99.6	+79.2	+53.7	+56.3
TLI	T1	−40.0	+40.0	−37.5	−77.5	−80.0	−2.5	+37.5
	T2	−322.5	−185.0	+57.5	−231.7	−137.5	−380.0	−90.8
	Mean	−181.3	−72.5	+10.0	−154.6	−108.8	−191.2	−26.7

## Data Availability

The data presented in this study are available upon request from the corresponding author. The data are not publicly available due to ethical restrictions.

## Abbreviations

The following abbreviations are used in this manuscript:

AUC	Area under the curve
AUCbc	Baseline-corrected area under the curve
CI	Confidence interval
HSI	Hyperspectral imaging
ICC	Intraclass correlation coefficient
ICC(3,1)	Two-way mixed-effects intraclass correlation coefficient for single measurements with absolute agreement
IgE	Immunoglobulin E
NIR	Near-infrared reflectance
OHI	Organ haemoglobin index
PoC	Proof of concept
RGB	Red, green, blue
ROI	Region of interest
SPT	Skin prick test
StO_2_	Tissue oxygen saturation
SWIR	Short-wave infrared
T1	Visit 1
T2	Visit 2
THI	Tissue haemoglobin index
TLI	Tissue lipid index
TWI	Tissue water index
